# Predictors for Long-Term Survival Free from Whole Brain Radiation Therapy in Patients Treated with Radiosurgery for Limited Brain Metastases

**DOI:** 10.3389/fonc.2015.00110

**Published:** 2015-05-11

**Authors:** Daniel Gorovets, Paul Rava, Daniel K. Ebner, David J. Tybor, Deus Cielo, Yakub Puthawala, Timothy J. Kinsella, Thomas A. DiPetrillo, David E. Wazer, Jaroslaw T. Hepel

**Affiliations:** ^1^Department of Radiation Oncology, Tufts Medical Center, Boston, MA, USA; ^2^Department of Radiation Oncology, Rhode Island Hospital, Providence, RI, USA; ^3^Department of Radiation Oncology, UMass Memorial Medical Center, Worcester, MA, USA; ^4^Department of Public Health and Community Medicine, Tufts University School of Medicine, Boston, MA, USA; ^5^Department of Neurosurgery, Rhode Island Hospital, Providence, RI, USA

**Keywords:** brain metastases, radiotherapy, gamma knife, radiosurgery, prognosis, survival analysis

## Abstract

**Purpose:**

To identify predictors for prolonged survival free from salvage whole brain radiation therapy (WBRT) in patients with brain metastases treated with stereotactic radiosurgery (SRS) as their initial radiotherapy approach.

**Materials and methods:**

Patients with brain metastases treated with SRS from 2001 to 2013 at our institution were identified. SRS without WBRT was typically offered to patients with 1–4 brain metastases, Karnofsky performance status ≥70, and life expectancy ≥3 months. Three hundred and eight patients met inclusion criteria for analysis. Medical records were reviewed for patient, disease, and treatment information. Two comparison groups were identified: those with ≥1-year WBRT-free survival (*N* = 104), and those who died or required salvage WBRT within 3 months of SRS (*N* = 56). Differences between these groups were assessed by univariate and multivariate analyses.

**Results:**

Median survival for all patients was 11 months. Among patients with ≥1-year WBRT-free survival, median survival was 33 months (12–107 months) with only 21% requiring salvage WBRT. Factors significantly associated with prolonged WBRT-free survival on univariate analysis (*p* < 0.05) included younger age, asymptomatic presentation, RTOG RPA class I, fewer brain metastases, surgical resection, breast primary, new or controlled primary, absence of extracranial metastatic disease, and oligometastatic disease burden (≤5 metastatic lesions). After controlling for covariates, asymptomatic presentation, breast primary, single brain metastasis, absence of extracranial metastases, and oligometastatic disease burden remained independent predictors for favorable WBRT-free survival.

**Conclusion:**

A subset of patients with brain metastases can achieve long-term survival after upfront SRS without the need for salvage WBRT. Predictors identified in this study can help select patients that might benefit most from a treatment strategy of SRS alone.

## Introduction

Brain metastases affect approximately one-third of all cancer patients (1). With recent improvements in life-prolonging systemic therapies, the incidence of brain metastases is increasing (2). Although the estimated median survival for all patients with brain metastases is only 4–6 months, there is substantial variability in prognosis and a subset of patients enjoy survival times well beyond 1 year (3, 4).

The appropriate upfront radiotherapy approach for newly diagnosed brain metastases is currently controversial with options that include whole brain radiation therapy (WBRT), stereotactic radiosurgery (SRS), or both (5–9). Given the potential late neurocognitive effects associated with WBRT, it would be particularly attractive to avoid it in patients with longer life expectancies (10–13).

Approximately 40–60% of patients with brain metastases treated with upfront SRS alone experience regional failures, some of whom require WBRT for salvage (5–7). Several tools have been developed to estimate survival; however, there are no available methods that predict which patients are likely to achieve long-term survival without the need for salvage WBRT (3, 4, 14, 15). It is this group of patients that would have the greatest benefit from an initial approach of SRS alone. The aim of this study is to identify patient, disease, and treatment variables that are associated with prolonged survival free from salvage WBRT.

## Materials and Methods

In compliance with institutional review board approval, the records of patients with brain metastases treated with SRS at our institution between 2001 and 2013 were reviewed. Patients who received prior or concurrent WBRT were excluded from this analysis. Patients who underwent surgical resection followed by SRS to the resection bed were included. The rates of local failure, distant CNS failure, salvage treatments, and overall survival were evaluated. Among eligible patients (*N* = 308), two groups with the most divergent outcomes were selected for comparison. The first group was defined as patients who survived and did not require salvage WBRT for at least 1 year following SRS (*N* = 104). The second group consisted of patients with poor outcomes, which was defined as patients who either died or required salvage WBRT within 3 months of SRS (*N* = 56). Patient, disease, and treatment variables were compared between these two groups.

### Steriotactic radiosurgery procedure

Patients selected for SRS alone at our institution had one to four brain metastases, Karnofsky performance status (KPS) ≥70, and life expectancy ≥3 months. Occasionally, patients were found to have additional occult metastases at the time of SRS and >4 lesions were treated with SRS alone. SRS was performed using a Leksell Gamma Knife Model C (Elekta, Inc., Stockholm, Sweden). Target lesions were identified using high-resolution magnetic resonance imaging (MRI) with intravenous gadolinium contrast. The target volume included the contrast-enhancing lesion with a 1–2 mm margin. Dose was prescribed based on tumor size according to Radiation Oncology Therapy Group (RTOG) study 90–05 (16). The median dose to the tumor margin was 20 Gy (range 14–22) generally prescribed to the 50% isodose line. Lesser margins and/or lower doses were used when tumors were near the brainstem or other sensitive structures.

### Outcomes assessment

Patients were seen in follow-up approximately 1 month after their SRS procedure and every 3 months following their initial post-procedure visit. MRIs were obtained at each scheduled follow-up visit. Local failure was defined as an enlarging, contrast-enhancing lesion on follow-up MRI at the site of SRS treatment. MR spectroscopy, MR perfusion analysis, and/or biopsy were performed when necrosis was suspected. Distant CNS failure was defined as a new contrast-enhancing lesion outside of the SRS treatment volume. Salvage WBRT was recommended for patients with distant CNS failure who had >4 new lesions, KPS <70, or predicted life expectance of <3 months.

### Patient, disease, and treatment characteristics

Patient, disease, and treatment data were collected by reviewing medical records. Patient data included age, gender, race, smoking status, cancer diagnosis, and histology. Disease variables included presence of neurological symptoms, number and location of brain metastases, size of the largest brain metastasis, and the presence and status of extracranial metastatic disease. Controlled systemic disease was defined as stable or responding disease on most recent imaging prior to SRS. Patients who were treated at initial cancer diagnosis were considered to have stable disease as they have not had an opportunity for systemic therapy. Oligometastatic disease was defined as the presence of ≤5 metastatic lesions (CNS included), whereas widespread disease was defined as >5 metastatic lesions (17–19). RTOG recursive partitioning analysis (RPA) classification was also determined for each patient (3). Treatment variables included surgical resection and SRS prescription dose.

### Statistical analyses

Actuarial local and distant CNS failures and overall survival were calculated using the Kaplan–Meier method. Age distribution, number of brain metastases, and size of brain metastases were evaluated by two-tailed *t*-tests with Welch’s correction. Chi-square or Fisher’s Exact Tests were used to compare groups based on gender, race, smoking status, neurological symptoms, RPA class, prior surgical resection, location of brain metastases, primary histology, primary disease status, and extracranial disease status. Based on the independent variables that were significantly associated with 1-year WBRT-free survival on univariate analysis, a multivariate binary logistic regression analysis was performed. Continuous variables were converted to binary variables. RPA class was excluded from the multivariate analysis because of collinearity with multiple independent variables (i.e., age and disease burden/control). A *p*-value of <0.05 was considered statistically significant.

## Results

**Figure 1:**
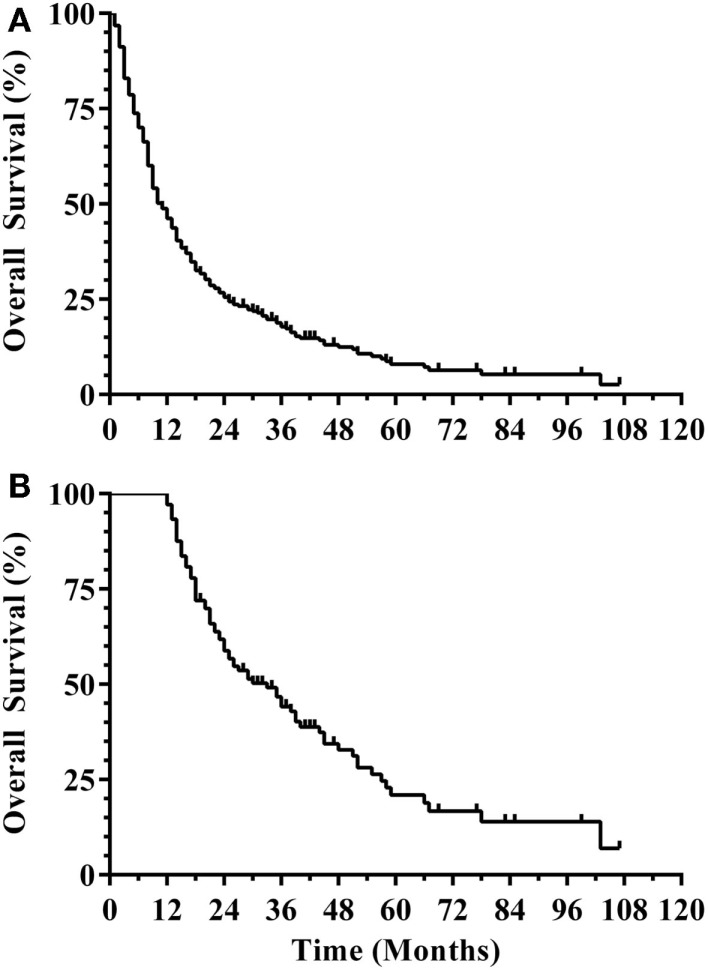
**(A)** Survival curve for all patients (*N* = 308). **(B)** Survival curve for ≥1 year WBRT-free survivors (*N* = 104) (WBRT, whole brain radiation therapy).

**Figure 2:**
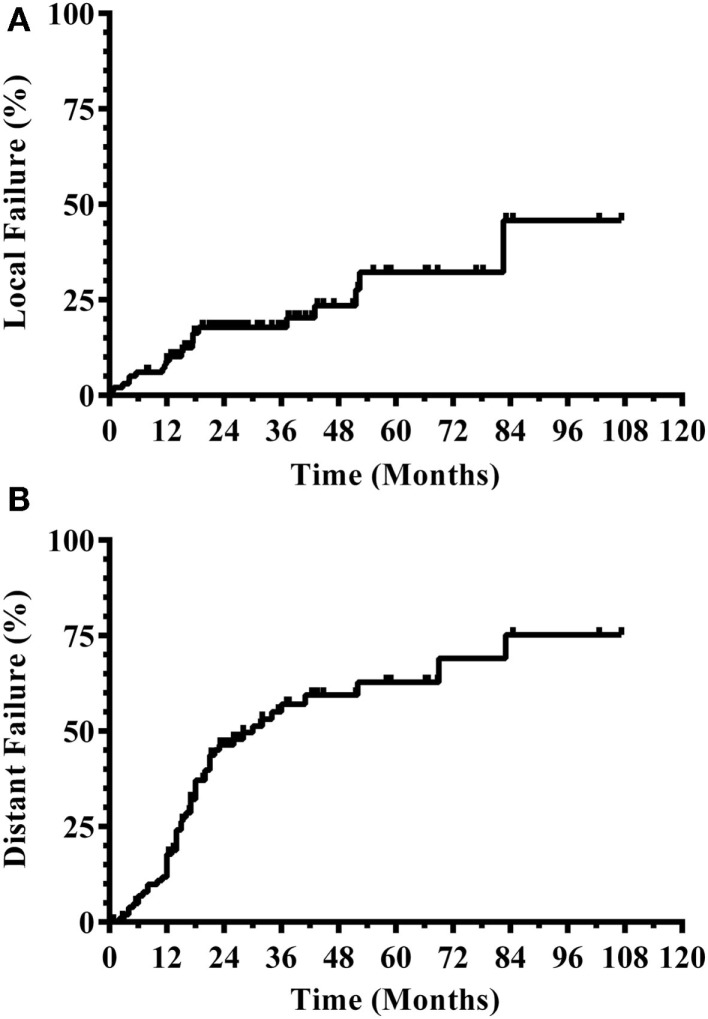
**CNS disease outcomes for patients with ≥1 year WBRT-free survival**. **(A)** Local progression. **(B)** Distant CNS progression (CNS, central nervous system; WBRT, whole brain radiation therapy).

Three hundred and eight patients were treated with SRS alone and fit inclusion criteria for analysis. The median overall survival was 11 months. The median WBRT-free survival was 8.5 months (range 0.8–107.3 months) with 30% ultimately requiring salvage WBRT. One hundred and four patients (34%) survived beyond 1 year without the need for salvage WBRT, while 56 patients (18%) either died or required WBRT within 3 months. Figure 1 shows the survival curve for the entire group of 308 patients treated with SRS alone and the survival curve for the 104 patients that form the subgroup with ≥1 year WBRT-free survival. The median overall survival in the group of patients with ≥1 year WBRT-free survival was 33 months (range 12–107.3 months) with 10% surviving longer than 5 years. Local failures occurred in 24% with an actuarial local control of 90.9% at 1 year and 82.2% at 2 years (Figure 2). Distant CNS failures occurred in 49% with 1 and 2 year actuarial rates of 17.8 and 46.4%, respectively. The median time to first intracranial salvage treatment was 19 months. Salvage treatments consisted of surgical resection in 7%, SRS in 42%, and WBRT in 21%.

Table 1 shows the patient, disease, and treatment characteristics of patients with ≥1 year WBRT-free survival and those with ≤3 months WBRT-free survival. Those with prolonged WBRT-free survival were significantly younger (*p* = 0.008), but were otherwise similar in regards to gender and race. Patients with favorable WBRT-free survival were also more likely to be asymptomatic at presentation (*p* = 0.029) and belong to RPA class I (*p* < 0.001). The patients who died or required WBRT within 3 months of SRS had more brain lesions treated at the time of initial SRS (*p* < 0.001). The mean size of the largest brain metastasis was not different between the two groups, but the patients with prolonged WBRT-free survival were more likely to have their largest brain metastasis surgically resected prior to SRS (*p* = 0.006). The comparison groups had similar SRS doses prescribed for the patients’ largest metastases. The majority of patients in both groups had lung cancer primaries, but the prolonged WBRT-free survival group had a larger proportion of breast cancer patients (*p* = 0.048), while the group with poor outcomes had a larger proportion of melanoma patients (*p* = 0.039). Patients with prolonged WBRT-free survival were more likely to have their cancer diagnosis coincident with the diagnosis of brain metastases or have a pre-existing cancer diagnosis with a controlled primary (*p* = 0.007). Extracranial metastatic disease was more common in the patients who died or required WBRT within 3 months of their SRS procedure (*p* < 0.001). Total disease burden was characterized as oligometastatic in 76.9% of patients with ≥1 year WBRT-free survival, while 60.7% of patients with ≤3 months WBRT-free survival had widespread disease (*p* < 0.001).

**Table 1:** **Patient, disease, and treatment variables stratified by WBRT-free survival group**.

	≥1 year WBRT-free survivors *N* = 104	≤ 3 months WBRT-free survivors *N* = 56	*p*-Value
Age, mean [range]	60 year [30, 83]	66 year [32, 89]	**0.008**
Gender, *N* (%)
Male	43 (41%)	31 (55%)	0.063
Female	61 (59%)	25 (45%)	
Race, *N* (%)
Caucasian	95 (91.3%)	48 (85.7%)	0.223
African American	1 (1.0%)	4 (7.1%)	
Hispanic	6 (5.8%)	2 (3.6%)	
Other	2 (1.9%)	2 (3.6%)	
Smoking status, *N* (%)
Active	23 (22.1%)	14 (25.0%)	0.263
Former	42 (40.4%)	22 (39.3%)	
Never	21 (20.2%)	16 (28.6%)	
Unknown	18 (17.3%)	4 (7.1%)	
Neurological symptoms, *N* (%)
Yes	72 (69.2%)	44 (78.6%)	**0.029**
No	29 (27.9%)	7 (12.5%)	
Unknown	3 (2.9%)	5 (8.9%)	
RTOG RPA, *N* (%)
Class I	40 (38.5%)	6 (10.7%)	**<0.001**
Class II	61 (58.7%)	48 (85.7%)	
Unknown	3 (2.9%)	2 (3.6%)	
Number of brain mets [mean (range)]	2 [1–9]	4 [1–13]	**<0.001**
CNS disease burden, *N* (%)			
Single met	64 (61.5%)	16 (28.6%)	**<0.001**
Multiple mets	40 (38.5%)	40 (71.4%)	
Size of largest met [mean diameter (range)]	2 cm [0.3–5.4]	2 cm [0.6–3.8]	0.762
Minimum dose [mean (range)]	19 Gy [14, 22]	18 Gy [14, 22]	0.149
Prior surgical resection, *N* (%)	30 (28.8%)	6 (10.7%)	**0.006**
Primary Histology, *N* (%)			
NSCLC	60 (57.7%)	29 (51.7%)	0.508
Breast	16 (15.4%)	3 (5.4%)	**0.048**
Melanoma	5 (4.8%)	8 (14.3%)	**0.039**
Other	22 (21.1%)	16 (28.6%)	0.158
Primary Status, *N* (%)
New/controlled	86 (82.6%)	35 (62.5%)	**0.007**
Progressive	9 (8.7%)	13 (23.2%)	
Unknown	9 (8.7%)	6 (10.7%)	
Extracranial mets, *N* (%)			
Yes	28 (26.2%)	37 (66.1%)	**<0.001**
No	67 (64.4%)	11 (19.6%)	
Unknown	9 (8.7%)	6 (10.7%)	
Total disease burden, *N* (%)			
Oligometastatic	80 (76.9%)	17 (30.4%)	**<0.001**
Widespread	14 (14.4%)	34 (60.7%)	
Unknown	9 (7.7%)	5 (8.9%)	

*Bold font indicates p < 0.05*.

*WBRT, whole brain radiation therapy; RTOG, Radiation Therapy Oncology Group; RPA, recursive partitioning analysis; met, metastasis; CNS, central nervous system; NSCLC, non-small cell lung cancer*.

The variables significantly associated with favorable WBRT-free survival identified by the univariate analyses were further evaluated with a multivariate binary logistic regression analysis (Table 2). When controlling for covariates, independent predictors of favorable WBRT-free survival were asymptomatic presentation, breast primary, single brain metastasis, absence of extracranial metastases, and oligometastatic disease burden.

**Table 2:** **Multivariate analysis comparing ≥1 year WBRT-free survivors vs. ≤3 months WBRT-free survivors**.

	Odds Ratio for ≥1 year WBRT-free survival^a^	*p*-Value
Age
<65 years vs. ≥65 years	1.81 [0.67, 4.88]	0.241
Primary histology
Breast vs. other	7.84 [1.34, 45.93]	**0.022**
Melanoma vs. other	0.59 [0.93, 3.77]	0.579
Primary disease status
New/controlled vs. progressive	1.10 [0.36, 3.32]	0.871
Neurological symptoms
Present vs. absent	0.19 [0.06–0.63]	**0.007**
Resected brain met
Yes vs. no	2.93 [0.76–11.38]	0.120
Number of brain mets
Single vs. multiple	3.07 [1.06–8.4]	**0.038**
Extracranial mets
Present vs. absent	0.24 [0.08–0.70]	**0.009**
Disease burden
Oligometastatic vs. widespread	6.32 [2.00–19.99]	**0.002**

*Bold font indicates p < 0.05*.

*^a^Relative to ≤3 months WBRT-free survival*.

*WBRT, whole brain radiation therapy; met, metastasis*.

## Discussion

Patients with brain metastases represent a very heterogeneous population. The optimal treatment for these patients remains an area of controversy. Survival well beyond 1 year is observed in a notable subset. For these patients, aggressive treatment to control their CNS disease is warranted.

Stereotactic radiosurgery alone has been adopted by many to treat patients with limited brain metastases and expected survival ≥3 months. Using this approach, distant CNS failures in approximately 50% are expected (5–7). To improve CNS control, others have adopted a combined approach of SRS and WBRT (8, 9). For the group of patients with potential for long-term survival, late treatment-related neurocognitive morbidity of WBRT must be considered (10–13). Three randomized phase III trials have evaluated SRS alone compared with WBRT and SRS (5–7). WBRT was shown to decrease the risk of distant CNS failure by 15–22%. However, a meta-analysis of these trials showed no survival advantage with the addition of WBRT to SRS; rather, in patients ≤50 years of age, WBRT was associated with increased mortality (20). Additionally, given the potential acute and late side effects of adding WBRT, it would be ideal to identify a patient cohort that is likely to achieve prolonged survival and yet unlikely to gain benefit from WBRT.

Several nomograms have been developed to predict survival in patients with brain metastases. Gaspar et al. performed a RPA using a database from RTOG trials with 1,200 patients and identified three prognostic groups (3). The median survivals for RPA class I (KPS ≥70, controlled primary, age <65 years, brain metastasis only), class II (not meeting criteria for classes I or III), and class III (KPS <70) were 7.1, 4.2, and 2.3 months, respectively. A more recent analysis seeking to better stratify patients is the disease-specific graded prognostic assessment (DS-GPA) (4). The RPA and DS-GPA scoring systems were developed based on patients who were primarily treated with WBRT and not with SRS alone. Two other groups have developed predictive models using large databases of patients treated solely with SRS (14, 15). Although the aforementioned prognostic systems were based on large numbers of patients and have been validated to estimate overall survival, they do not predict the likelihood of regional failure or the need for salvage WBRT.

Our study aimed to identify patient, disease, and treatment variables that might predict for patients who are likely to not only have prolonged survival, but also are unlikely to require salvage WBRT. We intentionally selected two groups of patients with the contrasting outcomes after SRS alone. Patients who lived beyond 1 year without WBRT had a median survival of almost 3 years, and only 21% eventually required salvage WBRT. It is important to note that the rate of salvage WBRT is dependent on the criteria used to offer salvage WBRT as compared with other salvage options such as repeat SRS. In our cohort, repeat SRS was offered only for patients with limited (≤4) new metastases and life-expectancy of at least 3 months, the same criteria as primary treatment. Patients not meeting these criteria were offered WBRT for salvage. Although some have advocated SRS alone for >4 and even >10 lesions, these patients suffer from a high rate of distant CNS failure (21, 22). A median time to distant CNS failure of only 3 months was seen in our experience treating patients with >10 lesions (23). We, therefore, have not offered routine salvage SRS for patients with >4 metastases.

In this study, predictors of ≥1 year WBRT-free survival on univariate analyses included younger age, RPA class I, breast primary, new or controlled primary, asymptomatic presentation, surgical resection of a brain metastasis, fewer brain metastases, absence of extracranial metastases, and oligometastatic disease. On multivariate analysis, asymptomatic presentation, breast primary, single brain metastasis, absence of extracranial metastases, and oligometastatic disease burden remained significantly associated with prolonged WBRT-free survival. Kondziolka et al. performed an analysis of 44 patients that survived longer than 4 years after SRS and compared them to patients that died within 3 months (24). They showed that patients with prolonged survival were significantly more likely to have fewer brain lesions, higher initial performance status, and less extracranial disease. In this study, however, 38 of the 44 patients received prior or concurrent WBRT. Kress et al. evaluated a cohort of non-small cell lung cancer patients treated with SRS alone to a single brain metastasis and showed that progression of systemic disease correlated with distant CNS failure (25). Ayala-Peacock et al. performed an analysis of 464 patients treated with SRS alone at Wake Forest University and evaluated variables predicting for distant CNS failure and need for salvage therapies (26). Histology (melanoma and HER2 negative breast cancer), progressive systemic disease, number of metastases (4–13 vs. 1–3), and occult metastases at time of SRS were found to be predictive of early time to distant CNS failure. Our data are consistent with these studies, showing that active disease burden, both in the brain and systemically, is the most influential predictor of WBRT-free survival.

The concept of oligometastatic disease was initially described by Hellman and Weichselbaum in 1995 (17). It is defined as a state between local-regional disease and wide-spread metastatic disease where metastases are few in number. In recent years, the oligometastatic disease state has gained much interest, as these patients have a more favorable prognosis compared to other stage IV patients (18, 19). In our study, oligometastatic disease was strongly associated with longer WBRT-free survival in both univariate and multivariate analyses. In the favorable group, over three quarters of the patients had oligometastatic disease compared to only one-third in the poor prognosis group.

Another interesting, but not surprising, finding from our analysis is the relationship between primary tumor type and WBRT-free survival. Multiple prior reports have demonstrated that compared to other histologies, overall survival outcomes are relatively better in patients with breast cancer (4, 27). Furthermore, recent data suggest that molecular subtypes of breast cancer are important in predicting survival, as well as CNS control after SRS alone (28, 29). Specifically, patients with HER2/neu over-expressing breast cancer with brain metastases have a lower risk of death and intracranial recurrence after treatment compared to patients with other breast cancer subtypes.

The interpretation of our results is limited by the retrospective nature and non-randomized study design, which introduces multiple well-described biases in data collection and analysis (30). Despite these potential shortcomings, using our institution’s guidelines for patient selection (≤4 CNS metastases and expected survival >3 months), 82% survived >3 months without requiring salvage WBRT and 34% survived >1 year without requiring WBRT. The results from our multivariate analysis suggest that patients who have oligometastic disease, controlled systemic disease, a breast primary and/or asymptomatic brain disease are expected to have favorable outcomes after SRS alone. Similarly, patients with widespread disease might be more optimally managed with the upfront incorporation of WBRT. Although a larger, prospective cohort of patients needs to be studied to confirm these findings, the identified predictive variables can be used to compliment and improve multi-disciplinary decision-making.

## Conclusion

Our results identify several variables related to intracranial and systemic disease burden that can help select patients that are likely to achieve prolonged survival and less likely to require salvage WBRT. This group of patients is ideally suited for SRS alone as their upfront radiotherapy approach. Further validation of these variables in a prospective cohort of patients is needed.

## Conflict of Interest Statement

The authors declare that they have no actual or potential conflicts of interest. No payments or services were received from a third party for any aspect of the submitted work. No financial relationships with entities that could be perceived to influence, or that give the appearance of potentially influencing, the work submitted need to be declared. No other relationships or activities that readers could perceive to have influenced, or that give the appearance of potentially influencing, the submitted work needs to be declared.
